# Cardiac dose reduction during tangential breast irradiation using deep inspiration breath hold: a dose comparison study based on deformable image registration

**DOI:** 10.1186/s13014-015-0573-7

**Published:** 2015-12-30

**Authors:** Ji Hyeon Joo, Su Ssan Kim, Seung Do Ahn, Jungwon Kwak, Chiyoung Jeong, Sei-Hyun Ahn, Byung-Ho Son, Jong Won Lee

**Affiliations:** Department of Radiation Oncology, Asan Medical Center, University of Ulsan College of Medicine, 88 Olympic-ro 43-gil, Songpa-gu, Seoul, 138-736 Republic of Korea; Department of Surgery, Asan Medical Center, University of Ulsan College of Medicine, Seoul, Korea

**Keywords:** Breast radiotherapy, Deep inspiration breath hold, Heart dose, Cardiac toxicity, Deformable image registration

## Abstract

**Background:**

Radiation therapy (RT) for a left-sided breast cancer often involves some incidental exposure of the heart and increase in the rate of major coronary events. One method to reduce the dose to the heart during a tangential breast irradiation is the deep inspiration breath hold (DIBH) technique. Our department adopted DIBH for selected left breast cancer patients with a maximum cardiac distance ≥ 10 mm. We evaluated the effect of the DIBH on cardiac dose compared to normal free breathing (FB). The secondary objective of our present study was to use modeled risk estimates to quantify the risk of coronary events after RT with DIBH.

**Methods and materials:**

Thirty-two patients who underwent RT with DIBH at our hospital were retrospectively analyzed. For each patient, two computed tomography (CT) scans were acquired, FB-CT and DIBH-CT. Using a deformable image registration tool, the target volume was deformed from DIBH-CT to FB-CT, and conventional tangential treatment planning was performed, focusing on the equality of target coverage between the two plans. Doses to the heart, left anterior descending (LAD) artery, and ipsilateral lung were assessed.

**Results:**

By using DIBH, the average mean heart dose was reduced from 724.1 cGy to 279.3 (*p* < 0.001). The relative heart volume irradiated with 10 Gy–50 Gy was consistently reduced. The mean dose to the LAD coronary artery was reduced from 4079.1 cGy to 2368.9 cGy (*p <* 0.001). The ipsilateral lung volume receiving 20 Gy or more and 40 Gy or more was reduced by 2.2 % in both cases. Estimated risks of coronary events at 10 years were 4.03 and 2.55 % for RT with FB and DIBH, respectively (*p <* 0.001).

**Conclusions:**

The use of DIBH during RT of the left-sided breast considerably reduces the doses delivered to the heart and LAD artery with similar target coverage. For the current study patients, the probability of major coronary events was reduced with DIBH.

## Background

Postoperative radiotherapy (RT) for breast cancer patients has been widely used since it was proven to reduce the risk of local recurrence and improve long-term survival [1, 2]. However, the absolute benefit of RT on long-term overall mortality is less than its benefit on breast cancer-related mortality. The excess mortality in these patients is mainly due to heart disease and lung cancer [1].

RT for left-sided breast cancer often involves some incidental exposure of the heart and ipsilateral lung to radiation. Exposure of the heart to radiation is related to increased rates of coronary events and cardiac mortality [3]. In tangential RT of left-sided breast, part of the anterior heart, including the left anterior descending artery (LAD), usually receives a non-negligible dose. It is not clear whether this results in myocardial damage or coronary artery damage, or both. Generally, the mean cardiac dose causes a proportional increase in the rate of major coronary events per gray. Darby et al. [3] have reported that the rates of major coronary events increased by 7.4 % per gray.

In recent years, considerable effort has been exerted to identify techniques that reduce the dose to the heart in patients receiving postoperative RT for breast cancer. One method involves respiratory gating using the deep inspiration breath hold (DIBH) technique. During deep inspiration, the heart moves posteriorly and inferiorly due to lung expansion and diaphragmatic movements. Thus, the distance between the chest wall and heart is maximized at or near the deep inspiration. Treatment delivery only at deep inspiration reduces the area of the heart that receives a high dose. Exposure of the ipsilateral lung to irradiation is not consistent with DIBH but the relative volume of the irradiated lung usually decreases. Several groups have reported a reduction in the heart and lung dose using DIBH in both dose planning and clinical studies [4, 5].

Our department adopted DIBH in selected left breast cancer patients with a maximum cardiac distance of ≥ 10 mm. Every patient who was treated using DIBH in our department underwent additional computed tomography (CT) with normal breathing (free-breathing CT [FB-CT]) in the same treatment position. We conducted our current CT planning study using two CT sets (DIBH-CT and FB-CT) in the same patient. When comparing the dose to organs at risk, it is essential that the target coverage is as equal as possible. The female breast is usually defined by an external surface structure. Generally, it can extend from mid-axillary line to mid-sternum, and from the second anterior rib to the sixth anterior rib. Because the shape varies depending on the position, it is not reliable to compare target coverage when the position of patient is different. During full inspiration, the shape of the breast tissue changes as the chest wall inflates and moves anterocranially. Thus, we used a deformable image registration tool to achieve the most consistent dose coverage.

Darby et al. [3] have previously conducted a population-based case–control study that suggested a reliable prediction model of major coronary events based on individual cardiac risk factors and the irradiated cardiac volume. Based on this model, we tried in our present study to predict the absolute risk reduction in women who were treated using RT after breast cancer surgery.

## Methods

### Patients

This analysis was conducted in accordance with the regulations of the Asan Medical Center Institutional Review Board (2015–0855). Between 2012 and 2015, 32 patients who were referred for adjuvant RT after left breast cancer surgery, including a breast-conserving operation or mastectomy, were treated using the DIBH technique at our institution. Patients were not consecutively included. After FB-CT, physicians decided whether to use DIBH. Principally, patients with a maximum cardiac distance ≥ 10 mm in a CT simulation, and with a good performance who could reproduce a breath-holding status were included. The mean age of the patients in our current study was 48 years (range, 25–77 years) and they also had to be able to hold their breath. All patients were treated using respiratory gating. Patient and treatment characteristics are summarized in Table 1.

**Table 1:** Patient, tumor, and treatment characteristics

Characteristics	*N* = 32 (%)
Median age at treatment (years)	48 (25–77)
Cigarette smoking	
Yes	1 (3)
No	31 (97)
Cardiopulmonary comorbidities	
None	19 (59)
Hypertension	7 (22)
Hyperlipidemia	2 (6)
Diabetes mellitus	4 (3)
Breast cancer stage	
I	9 (28)
II	9 (28)
III	14 (44)
Tumor stage	
T1	14 (44)
T2	11 (34)
T3	7 (22)
Nodal stage	
0	11 (34)
1	9 (28)
2	2 (6)
3	10 (31)
Breast conservation	
Yes	20 (63)
No	12 (37)
Systemic therapy	
Neoadjuvant chemotherapy	15 (47)
Adjuvant chemotherapy	13 (41)
Adjuvant trastuzumab	11 (34)
Adjuvant endocrine therapy	9 (28)
None	0 (0)

### Respiratory gating

The Varian Real-time Position Management respiratory gating system (version 1.7.5; Palo Alto, CA) was used for respiratory gating. An infrared reflecting marker was placed on the patient, normally over the xiphoid process, and its position was marked on the patients’ skin so that it could be reproduced during the treatment period. The anteroposterior motion of the marker due to chest wall movement was detected by a camera. Before CT simulation and delivery of the first treatment, patients were trained how to breath. No form of audio or visual guide was used but the operator told the patient when to take a deep inspiration and when to release their breath. The gating window was individually set to the maximum inspiration ± 5 mm. All respiration cycle curves were observed during whole treatment time by 2 experienced therapists and a physician. As the beam delivery was stopped by instant changes of the graph, the actual gating window was much smaller.

The continuous cine-image was further checked using an electronic portal image device (7.5 frames/s). Normally, a single tangential field took about 10 to 15 s. The CT slice thickness was 5 mm and the image acquisition was conducted in helical mode. Image acquisition commenced when the breathing amplitude marker reached the gating window. The scanning time was approximately 20 s and most patients managed to complete the scan during one respiration cycle.

### Delineation of the target and organs at risk

Contouring of the target volumes and organs at risk was performed in accordance with the guidelines published by the Radiation Therapy Oncology Group (RTOG) and the Danish Breast Cancer Cooperative Group (DBCG) [6, 7]. For breast-conserving operations, the clinical target volume (CTV) included all residual mammary tissues and was bordered by the clavicular head cranially, 20 mm inferiorly to the breast fold caudally, the mid-sternal line medially, and the mid-axillary line laterally. For mastectomies, the caudal margin was defined based on palpation of the contralateral breast.

Mammary tissue is usually visible on CT slices but there can be considerable inter- or intra-observer variability. Sometimes also, defining mammary tissues is confusing even for experienced radiation oncologists if, for example, the patient has very small breasts with loose breast tissues. To account for these uncertainties and ensure objectivity in our analysis, deformable image registration was used for the CTV. Using a contouring deformable registration tool (Mirada RTx Advanced ver. 1.62), the CTVs were deformed from DIBH-CT to FB-CT. Registered target volumes were checked by an experienced radiation oncologist. After deformable image registration, the planning target volume (PTV) was made separately in FB-CT and DIBH-CT, for beam generation and plan evaluation. The PTV was generated using a 5 mm margin from CTV, limited to the midline, and shrunk 5 mm from the skin. The PTV was not deformed because deformation of slit-like shaped PTV made it fragmented or distorted.

The ipsilateral lung was contoured using an automatic contouring tool. The heart was contoured in accordance with RTOG 1106 organs at risk atlases. Along with the pericardial sac, the superior aspect began at the level of the inferior aspect of the pulmonary artery passing the midline and extended inferiorly to the apex of the heart [8]. The LAD coronary artery was delineated in the anterior interventricular groove from its initiation down to the apex of the heart.

### Treatment planning

Two opposing 6 MV tangential conformal fields with a multileaf collimator were used. The prescription dose was 50 Gy in 2 Gy fractions. A minimum of 95 % of the target volume was to be covered by the 95 % isodose line (V95 ≥ 95 %). The tumor bed boost was usually delivered using a 3D-conformal plan, at 10 Gy in 2 Gy fractions, but this was excluded from our current analysis. In our institution, internal mammary node (IMN) irradiation is only considered in cases with a clinically suspected or pathologically proven metastasis. IMN irradiation was not done in any of the analyzed patients in this study. All treatment planning was performed by an experienced radiation technologist. The field-in-field technique and wedges were used to avoid hotspots exceeding 110 %. The dose to normal organs was kept as low as possible without compromising the target volume dose. For each patient, only minor differences between the two plans, with respect to target coverage, field-in-field, wedges, beam energy, and geometry, were accepted.

### Individual cardiac risk estimation

To estimate each individual's risk of major coronary events (myocardial infarction or coronary death), the Framingham Coronary Heart Disease Risk Score [9] and the assumption of Darby et al. (3) were used. The risk assessment tool from the Framingham Heart Study predicts the chance of myocardial infarction or coronary death in the next 10 years. It was chosen because RT-induced heart disease develops after a long period and the Darby model is based upon major coronary events. According to the Darby model, the excess risk of major coronary events is linearly dependent on the mean heart dose at a rate of 16.3 % during years 0–4 and 15.5 % during years 5–9 following breast RT. To estimate the risk at 10 years, we used the more conservative estimate of 15.5 % per gray because this likely approximates the excess relative risk during years 0–9 [10]. To group our current study patients according to their baseline risk, American Heart Association (AHA) risk groups were used. Patients were classified into high-risk, at-risk, or optimal-risk groups according to their risk factors [11].

### Statistical analysis

Dose–volume histograms were extracted and compared for each of the DIBH and FB plans. For the heart, the V_10Gy_–V_50Gy_, as well as the mean heart dose (D_mean_) and maximum heart dose (D_max_), were measured. For the left lung, the V_20Gy_, V_40Gy_, and D_mean_ were determined. For the LAD, the D_max_ and D_mean_ were determined. Paired t-tests were used for statistical analysis of the differences with SPSS statistical software version 21.0. Data were considered statistically significant at *p <* 0.05.

## Results

### Deformable image registration

**Fig. 1:**
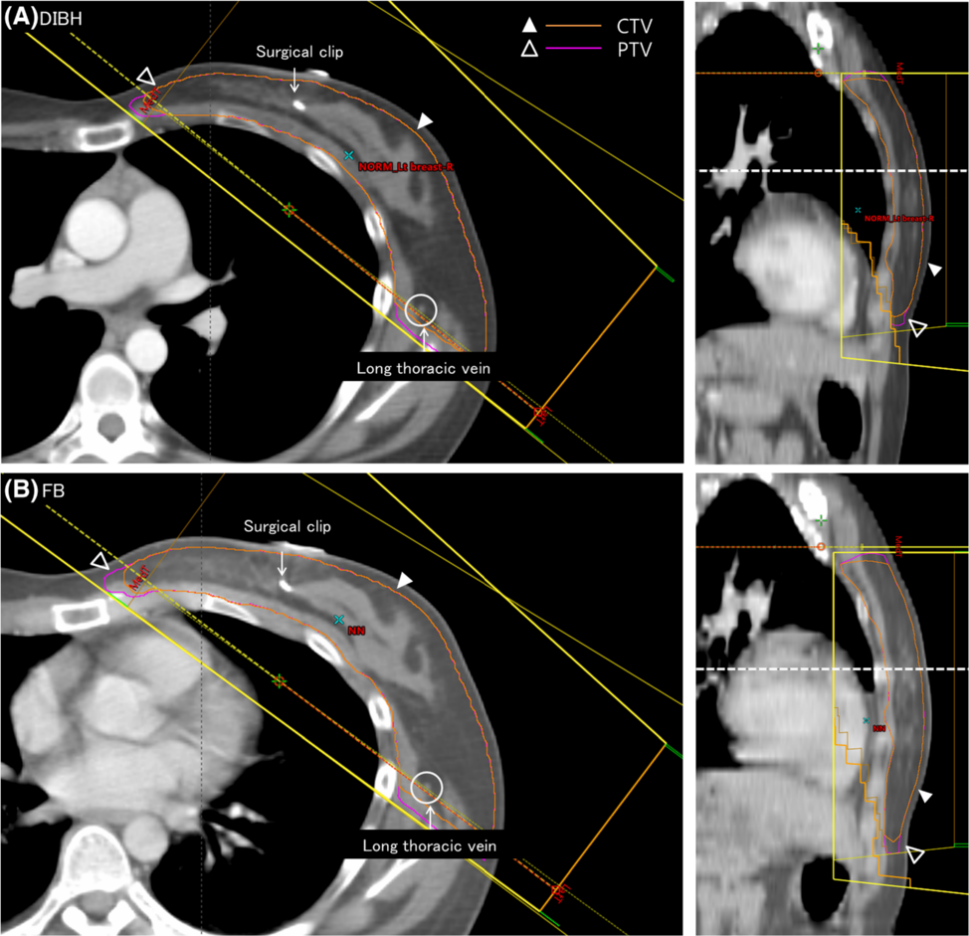
Axial computed tomography sections with deep inspiration breath holding (DIBH) (**a**) and free breathing (FB) (**b**) techniques. The anatomical matches of the CTV are confirmed by reference to the sternum and long thoracic vein. CTV, Clinical target volume; PTV, planning target volume

**Fig. 2:**
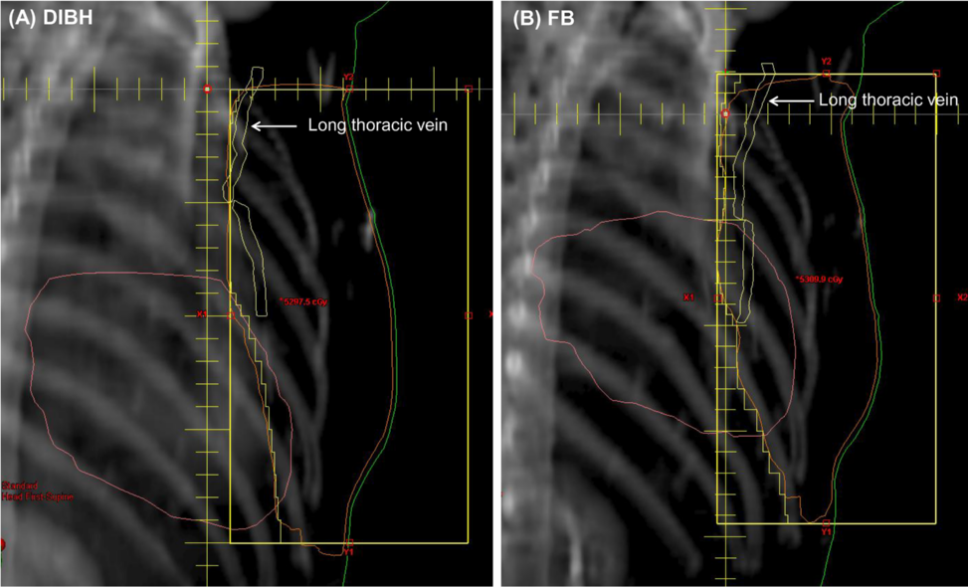
Digitally reconstructed radiographs of the deep inspiration breath holding (DIBH) (**a**) and free breathing (FB) (**b**) techniques. Posterior–inferior displacement of the cardiac apex and decreased maximal heart distance are shown during DIBH

In most of the current study cases, the anatomical correlation was excellent, and additional adjustment was not needed. The equality of target coverage was checked by measuring the shortest distance between the beam margin and long thoracic vein in digitally reconstructed radiographs (no significant difference). One example each of DIBH-CT and FB-CT is shown in Fig. 1. In the axial CT image, the deformed CTV is anatomically well correlated, as shown by its relation to the sternum and the long thoracic vein. The posterior–inferior cardiac displacement and decreased heart distance in the tangential field is shown in the sagittal section and digitally reconstructed radiograph (Fig. 2).

### Cardiac dose

The main results of the comparisons of the mean values of the dose metrics for the DIBH and FB plans are listed in Table 2. In the DIBH plans, 2 of the 32 hearts were outside the beam portal, whereas all hearts were included in the beam portal in the FB plans. The average maximal heart distance decreased from 2.1 cm (FB: range 1.2–3.9 cm) to 0.7 cm (DIBH: range, 0–1.6 cm) (*p <* 0.001). The average mean heart dose was also reduced, from 724.1 cGy (FB: range, 310.5–1405.3 cGy) to 279.3 cGy (DIBH: range 116.5–521.0 cGy) (*p <* 0.001). The relative heart volume irradiated with 10 Gy–50 Gy was consistently reduced by DIBH. The V_10_, V_20_, V_30_, V_40_, and V_50_ values for FB vs. DIBH were 14.6 vs. 4.0 % (-73 %), 12.3 vs. 2.7 % (-78 %), 10.7 vs. 2.0 % (-82 %), 8.7 vs. 1.3 % (-85 %), and 2.5 vs. 0.2 % (-91 %), respectively. The relative reductions in cardiac doses were similar between the low- and high-dose regions. The maximum heart dose was 5114.0 cGy with FB and 4947.4 cGy with DIBH (*p =* 0.191). In 26 patients (81 %), the V_40_ value was larger than 5 % when using the FB technique compared with 0 % (i.e. none of the patients) when using DIBH.

**Table 2:** Comparisons of dose metrics

		DIBH		FB		*p*-value
		Average	SD	Average	SD	
Heart	D_mean_ (cGy)	279.3	99.7	724.1	272.1	0.000
	D_max_ (cGy)	4947.4	810.6	5114.0	309.1	0.191
	V_10_ (%)	4.0	2.2	14.6	6.0	0.000
	V_20_ (%)	2.7	1.8	12.3	5.5	0.000
	V_30_ (%)	2.0	1.5	10.7	5.2	0.000
	V_40_ (%)	1.3	1.2	8.7	4.6	0.000
	V_50_ (%)	0.2	0.4	2.5	4.0	0.001
	MHD (cm)	0.7	0.4	2.1	0.6	0.000
Lung	D_mean_ (cGy)	943.7	223.2	1018.4	300.9	0.008
	V_20_ (%)	16.7	4.4	18.9	6.3	0.000
	V_40_ (%)	11.9	3.6	14.1	5.5	0.000
	CLD (cm)	2.9	0.6	2.6	0.5	0.014
LAD	D_mean_ (cGy)	2368.9	1162.1	4079.1	940.2	0.000
	D_max_ (cGy)	4720.9	911.8	5058.6	338.7	0.044

*DIBH* deep inspiration breath hold, *FB* free breathing, *SD* standard deviation

For the LAD coronary artery, the D_mean_ was significantly reduced from 4079.1 cGy (range 568.3–5359.3 cGy) to 2368.9 cGy (range, 370.0–4415.0 cGy) using DIBH (*p <* 0.001). The maximum LAD artery dose was 5058.6 cGy (range, 4389.8–5604.6 cGy) with FB and 4720.9 cGy (1505.9–5610.9 cGy) with DIBH (*p =* 0.010). Due to its anatomical position, the LAD coronary artery is often within the high-dose region. In 9 and 7 of the FB and DIBH plans, respectively, the maximum LAD artery dose was higher than 5000 cGy.

**Fig. 3:**
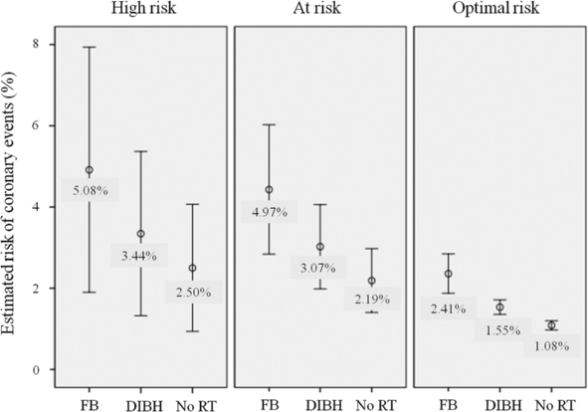
The 10-year estimated risk using the free breathing (FB) technique vs. deep inspiration breath hold (DIBH) technique vs. no radiation therapy (RT) according to the American Heart Association (AHA) risk grouping

The baseline and estimated coronary event risks were evaluable in all 32 study patients. About one-third of the patients had cardiopulmonary comorbidities (Table 1). From the plan comparisons, the estimated risks of coronary events at 10 years in all patients were 4.03 % (range, 1.48–21.74 %) and 2.55 % (range, 1.18–13.79 %) for RT with FB and DIBH, respectively (*p <* 0.001). The median 10-year relative risk reduction was 32 % (range, 16.15–83.28 %) with the DIBH technique, and the absolute risk reduction was 1.48 % (range, 0.25–7.95 %). The AHA risk grouping was high risk, at risk, and optimal risk in 4 (13 %), 16 (50 %), and 12 (37 %) patients, respectively. The 10-year estimated risks using FB vs. DIBH were 5.08 vs. 3.44 % in the high-risk, 4.97 vs. 3.07 % in the at-risk, and 2.41 vs. 1.55 % in the optimal-risk groups (Fig. 3).

### Pulmonary dose

The distance of the lung included in the beam portal increased when using the DIBH technique. The average central lung depth was 2.6 cm with FB and 2.9 cm with DIBH (*p =* 0.014). However, the average mean pulmonary dose was reduced with DIBH, from 1018.4 cGy (range 488.9–1516.8 cGy) to 943.7 cGy (range 505.1–1386 cGy) (*p =* 0.001). Similarly, for the average ipsilateral lung volume receiving 20 Gy or more, the V_20_, was reduced from 18.9 % (range 8.3–30.9 %) to 16.7 % (range 9.2–24.7 %) (*p <* 0.001). The V_40_ was also reduced from 14.1 % (range 5.8–23.8 %) to 11.9 % (range 6.3–19.2) with DIBH (*p <* 0.001). Although the lung depth in the beam portal was increased, all pulmonary dose parameters improved when using DIBH, due to lung expansion. Similar to the heart, the relative reduction in the exposed lung volume was similar between the low and high doses.

## Discussion

Assuming that the same dose coverage has been achieved, the heart and lung dose parameters were significantly reduced in the present study patients by using respiratory gating. The average heart distance in the tangential field was reduced from 2.1 cm to 0.7 cm, and the mean cardiac dose was reduced from 724 cGy to 279 cGy (-61 %). The reduction in the mean cardiac dose was similar to that of earlier studies but the absolute cardiac dose and range of dose reduction was higher in our current study. Swanson et al. [12] reported a mean heart dose reduction of 4.2 Gy with FB vs. 2.5 Gy (-40 %) with DIBH. All other heart parameters evaluated favored the delivery of DIBH over FB plans. Vikstrom et al. [5] showed an average mean dose of 3.7 Gy with FB for the heart and 1.7 Gy (-54 %) with DIBH. Stranzl et al. [13] reported a mean heart dose reduction of 2.3 Gy with FB vs. 1.3 Gy (-56 %) with DIBH. DIBH also shows benefit when combined to modern technique RT. In a study by Hayden et al. [14], DIBH resulted in a significant reduction in radiation dose to the heart and LAD compared with an FB, utilizing tangential intensity modulated RT (IMRT) plans with simultaneous integral boost. Nissen et al. [4] found that the mean heart dose reduced from 5.2 Gy to 2.7 Gy (-48 %) with a DIBH and IMRT plan. As the prescribed dose in all studies, including our current report, was 50 Gy/25 fx, there are two possible explanations for the discrepancy. First, not all left breast cancer patients were treated using DIBH at our center. We specifically included selected women with a maximum cardiac distance ≥ 10 mm in CT simulation. Second, due to the smaller lung volume of Asian women compared with Caucasians, the distance between the left ventricle and chest wall might be shorter [15, 16]. This is supported by the higher D_max_ of the heart in the DIBH plan (25–27 Gy vs. 49 Gy) and the lower proportion of patients (59 vs. 6 %) in our current series whose heart was completely outside the fields. Thus, we suggest that DIBH is particularly helpful for patients with a long maximal heart distance and small lung volume, such as Asian women.

With DIBH, we found that the relative reductions in cardiac doses were similar between low- and high-dose regions. For postoperative RT of breast cancer, the aspect that is most responsible for ischemic heart disease, for example, the mean dose or maximum dose of the heart or LAD artery, is still debated [17, 18]. In addition, the part of the heart anatomy most affected by radiation is still inconclusive. In a study by Correa et al. [19], a statistically significant higher prevalence of cardiac stress test abnormalities was seen among left-side irradiated patients vs. those of the right side (8 vs. 59 %). Moreover, most left-sided abnormalities in that study (70 %) were in the LAD artery territory. Mark et al. [20] found that RT causes volume-dependent perfusion defects in approximately 40 % of patients within 2 years of RT. The perfusion defects were significantly associated with the left ventricular volume included within the RT field. With these results, the target of the deleterious radiation in breast cancer treatment is thus the coronary artery. In a study by Nilsson et al. [21], a direct link between the RT field and location of coronary artery stenosis was observed. However, microvascular (fibrotic) damage is also possible after a longer latency period [22].

Although the mechanism is unclear, the relationship between the mean cardiac dose and cardiac disease risk is well established. Furthermore, the LAD artery dose closely correlates with the mean cardiac dose [23]. Thus, estimation of the risk of the radiation-related ischemic heart disease might be most appropriately derived from the cardiac mean dose. In our present study, the average reduction in the cardiac mean dose was 444.8 cGy. The estimated 10-year risks of coronary events were 4.03 and 2.55 % for our patients treated with FB and DIBH, respectively. Although the absolute risk reduction was relatively small (1.48 %), our current findings may be clinically significant given the high incidence of breast cancer and the high prevalence of long-term survivors. The estimated clinical benefit of DIBH was also studied previously by Eldredge-Hindy et al. [10]. The risk of ischemic heart disease was reduced with DIBH in that study, regardless of the baseline cardiac risk, although the largest benefit was observed in the high-risk group. Korreman et al. [24] used the relative seriality model to calculate the expected reduction in cardiac mortality from the use of DIBH in 16 cases. They found that the cardiac mortality probability was reduced from 4.8 % in FB to 0.1 % for DIBH.

Another strategy to minimize radiation exposure is the treatment position. In the prone position, the breast is elongated and falls away from the trunk. Studies comparing supine and prone whole-breast irradiation have shown that the prone position can reduce lung volume exposure [25]. A major disadvantage of the prone setup is the gravity-induced anterior displacement of the heart toward the field edge. To address the problem of a higher heart dose in the prone position, prone DIBH has been attempted. In a study by Mulliez et al. [26], four treatment plans—supine shallow breathing, supine DIBH, prone shallow breathing, and prone DIBH—were compared. The authors found that DIBH was able to reduce the heart dose in both positions, with the results of prone DIBH at least as favorable as those of supine DIBH. While preserving the lung-sparing ability of prone positioning, prone DIBH was able to reduce the heart dose compared with prone shallow breathing. To maximize normal tissue sparing, DIBH in the prone position could be considered, especially for young small-breasted patients who are able to perform prone DIBH.

There were some limitations to our present study. First, our analysis was limited to 32 nonrandom cases. However, it is likely that larger numbers of patients would show similar results. The strength of our current study was that exactly the same patient cohort was included in the FB and DIBH plans. Using the deformable image registration technique, the equality of treated mammary tissue was maintained. Also, both breast-conserving surgery and mastectomies were included in the analysis.

## Conclusions

DIBH allows tangential treatment of left-sided breast cancer patients and considerably reduced radiation doses to the heart, lung, and LAD artery without compromising target coverage. For our study patients, the probability of a major coronary event within 10 years was reduced from 4.03–2.55 %.
